# Contributions From Psychology to Effectively Use, and Achieving Sexual Consent

**DOI:** 10.3389/fpsyg.2020.00092

**Published:** 2020-02-19

**Authors:** Ramon Flecha, Gema Tomás, Ana Vidu

**Affiliations:** ^1^Department of Sociology, University of Barcelona, Barcelona, Spain; ^2^School of Law, University of Deusto, Bilbao, Spain

**Keywords:** sexual consent, social impact, communicative acts, sexual relationships institutional power, interactive power

## Abstract

Psychology related to areas such as gender, language, education and violence has provided scientific knowledge that contributes to reducing coercive social relationships, and to expanding freedom in sexual-affective relationships. Nonetheless, today there are new challenges that require additional developments. In the area of consent, professionals from different fields, such as law, gender, and education, are in need of evidence differentiating human communication that produces consent, and those conditions that coerce. Up to now, consent has been focused on verbal language, for example, “no means no,” or “anything less than yes is no.” Despite the fact that focusing consent on verbal language is a very important part of the problem, it does not solve most of the issues currently raised, like the famous case of “La Manada” in Spain. This article presents the most recent results of a new line of research, which places the problem and the solution in communicative acts, not only in speech acts. Even though there might be a “yes” in a sexual-affective relationship, there might not be consent, and it is indeed a coercive relationship if that “yes” has been given in a relationship determined by institutional power or by interactive power. Institutional power may occur if whoever made the proposal for the relationship is a person in charge of the process of selecting personnel in a company, and one of the candidates is the person who receives the proposal. Interactive power may occur if whoever makes the proposal is situated in an equal or inferior position in the company to the person receiving it, but the former threatens sextortion the latter. The potential social impact of this research has been already shown in the cases analyzed for this study.

## Introduction

According to a 2017 report from the World Health Organization (WHO), 35% of women worldwide have suffered some kind of physical or sexual violence throughout their lives. The World Health Organization [WHO] (2017) also state that interpersonal violence is one of the leading causes of deaths in women aged between 15 and 44 worldwide; ahead of deaths caused by cancer, wars or traffic accidents (World Health Organization [WHO], 2018). In addition, another gender-based-violence (GBV) related concern is the decreasing age of the victims. Almost 1 in 3 adolescents aged 15–19 years suffer or have suffered violence in their sexual-affective relationships (World Health Organization [WHO], 2018). This data leads us to consider GBV occurs from adolescence (Puigvert et al., 2019). Pressure from “friends” to have sporadic relationships or sexual encounters have, in some cases, led to the death of several girls who refused to continue the encounter at some point during the sexual contact.

Gang rapes are an increasingly present reality as recent media (Catalan News^1^; The Guardian^2^; BBC News^3^), statistics (Geoviolencia sexual^4^) and research (Dixon et al., 2019) has shown, and there is still little research dedicated to this phenomena. According to the Geoviolencia platform, gang rapes occurred in Spain increased from 18 in 2016, to 60 in 2018 (and counting 42 in the first half of 2019). Factors identified an intersection of several contextual elements to explain gang violence (Dixon et al., 2019). In the same way, authors claim the need for a bridge between interdisciplinary areas of study to better explain interpersonal violent crimes While studying gang-rape perpetuators, Porter and Alison (2019) determined the existence of leaders who are more influential in the offense, encouraging others to be implicated in the crime. Indeed, researchers have questioned the psychology of criminal conducts for decades (Fortune and Heffernan, 2018), and consider the need to socially approach criminal behaviors, involving both individuals and the community.

The analysis presented in this study by Puigvert et al. (2019), not only focuses on the problem related to the causes of gender-based violence, but also analyzes its underlying factors to identify effective actions that may contribute to prevent young girls and women from becoming victims. They observed that boys with violent attitudes and behaviors mostly preferred one night stands, while those boys with non-violent traits mostly preferred stable relationships. This separation between types of boys leads some girls to tend toward having a “a good time” with the more violent boy, and later in life to settle down with the other type of egalitarian boy (Gómez, 2014). These two models potentially lead to gender-based violence, and above all, it tends to force and intimidate girls, even at very early ages, to hook up and have sexual experiences that they may have not chosen on their own.

Duress and coercion also exist in relationships between teenagers. In a study conducted by Katz et al. (2019), 422 students between the ages of 15 and 16 were surveyedwith just one question: “Did someone with whom you are dating or with whom you dated, force you to do sexual things that you did not want to do?” The results showed that approximately 22% of women and 8% of men reported having experienced sexual coercion at least once in their life. Findings of this study illustrate that sexual coercion tends to be a common element among adolescents. Poor quality relationships, many of them based on this type of forceful friendship might have a long-lasting impact people’s lives. On the other hand, high quality relationships protect against harassment and ensure a good quality of life (Harvard Study of Adult Development^5^). Positive relationships also improve work environments and may contribute to overcome situations of conflict and violence (De Cordova et al., 2019).

All types of relationships, at any age, should be free of coercion. Social influence, especially peer influence, plays a crucial role on adolescent decision making (Ciranka and van den Bos, 2019). Education on consent is needed for all children, youth, teens and adults. People from all walks of life are participating with the feminist movement, and joining the struggle against sexual harassment to make to possible to have positive sexual relationships (Joanpere and Morlà, 2019). Awareness on consent, from early ages, has to do with freedom, shaping the limits of one’s own body and that of the other person. Duress, coercion and any similar kinds of acts committed by another individual, are considered harmful and they can potentially involve seriously adverse life-course health consequences (Bellis et al., 2019). Indeed, sexual harassment and gender-based violence have serious negative repercussions on people’s physical and mental health (World Health Organization [WHO], 2018). Kandel et al. from the neuroscientific field, also demonstrated GBV’s long-term effects on people’s health (Kandel et al., 2012). Considering it as a public health issue, the damage of violence is difficult to endure psychologically for many victims. Addressing this reality becomes a matter of high importance for psychology.

To properly tackle this scourge, society is at a crucial historical moment; not only from social movements such as #MeToo and similar, but also from research. The European Commission and its Directorate of Research has social impact as one of their priorities for the near future (Flecha et al., 2015). In line with this concerning problem of GBV and with the aim of improving people’s lives, the European Commission has designed 17 priorities within the agenda of its Sustainable Development Goals (SDGs), to carry out together with 169 associated targets. Goal 5: *Achieve gender equality and empower all women and girls* shows the EU commitment with Gender equality in line with European values rooted under the EU political and legal framework. “The EU’s strategy and action plan to promote *Gender equality* and women’s empowerment aims at changing the lives of girls and women by focusing on their physical integrity, promoting women and girls’ economic and social rights, their empowerment and strengthening their voices and participation^6^.” This statement also highlights the need and willingness of governments, professionals and policy-makers to act in this line, empowering women victims to make their voices heard, and to support them.

Different scientific research has already demonstrated the impact of interpersonal violence or aggression on the physical and mental health of people (Waldinger et al., 2006; Shonkoff et al., 2012). Indeed, gender-based violence may intersect with other inequalities (Shefer, 2019). Sexual justice and gender rights are becoming main aims for scholars and activists all over the world. Prevention interventions are also urgent and necessary, as a modifiable factor of risk, protection and above all rejection of any unconsented attitude. Contributing from psychology to effective use and achievement of sexual consent, examples of the most successful actions in this area will be analyzed in this article.

## State of the Art

Psychology as a discipline has contributed for decades to the research on gender, violence and someway on consent too (Walker, 1979; O’Connell and Russo, 1991; Jordan et al., 2010). From the studies of American psychologist Leonor Walker, leader in the field of domestic violence and founder of Domestic Violence Institute, analyzed psychological contributions on the social problem of men’s violence against women (VAW) based on power, and a kind of socialization of men who believe they may control women by any means (Walker, 1989). In this vein, we look at the necessity of a feminist analysis in psychology during the late 1960s, when the feminist movement began examining the gender role socialization on female and male behaviors, previously considered biologic and innate (Maccoby and Jacklin, 1974). Thus, this new psychological field was created focused on women and men power and their role in society (Dilling and Claster, 1985). In this sense, the issue of consent is raised from several perspectives, from the latest research in psychology, which is includes people’s ways of thinking, behavior and ways of interpreting other’s actions as well as the way consent is willing to be asked and provided. In the case studies taken as examples for this review article, it is easy to see how consent need to be free, agreed, informed, during all time and legislated under the basis of communicative actions.

Known as a major pioneers in the study of women in psychology, Agnes O’Connell and Nancy Russowrote on women’s life stories and heritage in the origins and development of psychology (1991). With this publication, celebrating the American Psychological Association’s Centennial, the scholars recognize eminent women and feminists who serve for the transformation of traditional psychological theories, methods and practice, aiming to preserve their contributions within an appropriate historical and sociocultural context (O’Connell and Russo, 1991). One of these traditional psychological theories consisted of blaming victims, for instance questioning their way of dressing, and without even considering rape at home, or by dating partners; linking their trauma with the need for therapy (Walker, 1989). Sexually abused children were following theories proposed by psychoanalysts such as Freud also blaming them, until the moment when feminist psychologists were finally able to discredit the myth of the seductive child (Lerman, 1986; Walker, 1988).

In the same line, research on psychology has other social impacts. Using psychological research methods to approach VAW, Walker’s research helped to develop programs for survivors to attend, to create new policies and change old legislation (Schneider, 1986; Walker, 1989). In terms of gender violence, psychology also conduced to point out that gender discrimination might contribute in making women vulnerable to potential mental health problems. As an expert on psychology and psychiatry, and founding director of the Center for Research on Violence Against Women at the University of Kentucky, Jordan et al. (2010) argues that, besides considering VAW as a legal and social justice problem, research should also focus on the psychological impact of violence on victims. New scientific psychology is integrating gender analysis when considering victim’s voices, case studies and basing new approaches on survivors’ narratives.

As described, much psychological research has contributed to the study of gender-based violence. Drawing on previous research, our contribution pretends to approach the use and achievement of sexual consent.

MacKinnon (1979) was one of the pioneer scholars, contributing to raise awareness about the importance of legislating what is considered sexual harassment in public institutions. MacKinnon aimed to achieve gender equality in international and constitutional law, as well as in political and legal theory. She also worked toward obtaining legislation against sexual harassment in the United States and other types of GBV that violates civil rights. Title IX itself constitutes an example of this struggle for legislation. Pretending to make gender bias visible into law, she began to open a legal debate on issues such as sexual discrimination and sexual abuse. MacKinnon (2005) supported the recognition of sexual harassment, rape and abuse based on power, focusing the point on affirmative consent, which led to reformulate the debate on United States legislations and gender equality.

Along this line, in a broader understanding of communicative sexuality (MacKinnon, 1983) other feminists have explored it further. Pineau (1989) is considered one of the first women who, analyzing legislation from a feminist point of view, examined victimization toward vulnerable women. She raised the point on rape based on non-consensual sex, and thus opened the debate on a more communicative sexuality model, so that consent should be explicit and clear, objective and legislated, with a more complete model that “no means no” or “yes means yes.” Similarly, Cowling (2004) also suggested a move toward teaching a communicative model of consent. Her research provides evidence that consent communication occurs most often indirectly and non-verbally.

In the European context, Wilson (2000) discusses the subjective experience of sexual harassment and assault. Based on data from university students in Scotland, Great Britain, New Zealand and North America, Wilson argues that for a correct analysis of the understanding of sexual harassment, the representation of both the complexity of thought and the behavior of someone who has suffered this harassment is necessary. These two spheres refer to the psychological impact that harassment triggers in people. Fiona Wilson claims the need to better understand individual experiences and how harassment or aggression produces some kind of labels in people’s lives. This subjective world, in terms of Habermas (1987), frames a world only accessible for individuals themselves. A deep understanding connecting harassment and its psychological effects is crucial to better defend its approach and legislation. In this sense, people and survivors’ narratives (Clark and Pino, 2016; Miller, 2019) have shed light on the victim’s healing, peer-support affect and a way of making their stories public. This achieves both solidarity with other survivors, an awareness raised of GBV, and the need to engage people into action.

Known as a representative of modern psychology, Bruner turned the discipline placing intersubjectivity and narrative at the center, pointing out that the future of psychological science was linked to understanding of the human mind in relation to human interaction and the cultural context (Bruner, 1996). As a social psychologist, G. Mead (1934) founded what is now called the symbolic interactionism. Mead describes a sociological perspective of interaction, how individuals interact with one another, in order to communicate and create “symbolic worlds,” while these “worlds” shape each individual’s behaviors. In this way, society as a whole is built through interactions in a continuous process of interpretation of people’s worlds and the meanings they share and develop among them.

Social and cultural factors form not only our environment and understanding but also our brain. By shaping our way of thinking and behaving in society, understanding and applying consent becomes singularly relevant. This is true for making each person understand that they own their bodies, and above all, for making people with whom interaction is produced, understand not only the impossibility of touching another body without permission, but the severity of doing so. Thus, individuals would then avoid memories of unwanted relationships, which leave deep marks on human brains (Hirst and Rajaram, 2014) and potential problems later in life. Within the challenges for free cognitive development, psychologists begin to pay attention to violence in its broadest aspect (Racionero-Plaza, 2018).

Many authors have researched for decades to find the most beneficial ways to intervene to support survivors or position themselves in the context of GBV at universities. The multi-longitudinal study of Coker et al. (2016) already shows that programs for overcoming and acting against harassment, based on bystander intervention are the most efficient ones. From the academic field, research on this topic has been extended to other areas. Recent studies in psychology (Philpot et al., 2019) continue to affirm the success of bystander intervention. In a comparative analysis between countries of different continents, this research demonstrates that in most public conflicts, the tendency of bystanders is to intervene to help someone in an emergency. People are also more likely to intervene when accompanied by other people. Based on these findings, Philpot et al. (2019) argue the need for psychology to change the narrative of the absence of help, toward a new understanding of what makes intervention successful.

Approaching consent also involves an important link between psychology and legislation framed within the difficulty of legislating people’s wills. Consent may be non-verbal and dependant on context. How the other person is willing to understand it as such, so as not to commit a crime, also depends on what is considered moral, correct, or legal and what is not. Slavery for instance, would be immoral today. Even just a few decades ago, many women did not have a say over their marriage, as consent would have been given by their parents or siblings. Since then, society has changed to give women this autonomy, and has encouraged them, with men, to continue the struggle for more rights. It is not acceptable to touch another body without permission, being it the most precious thing that makes us human beings; and so, laws have to legislate it to protect citizens. The most essential part of a human being cannot be assumed in any other way.

### Beyond Words Defining Consent and Asking for Its Regulation

According to the National Sexual Violence Resource Center (NSVRC, 2015^7^) consent is understood as “*an affirmative agreement to engage in various sexual or non-sexual activities. Consent is an enthusiastic, clearly communicated and ongoing yes. One can*’*t rely on past sexual interactions, and should never assume consent*” (NSVRC, 2015). The student movement was pioneering in opening the debate on consent in sexual relationships. In 2004, “Understanding consent to Sexual Activity” constituted one of the first laws known in the topic, making the “No means no” a pivotal slogan in this regard. The bill cements that states of unconsciousness, alcohol, and drugs, make someone unable to give consent. In addition, fear, intimidation, power relations, and academic evaluations are situations that may inhibit the victim’s capacity to say “no”; so consent should be nullified in this context.

“Affirmative consent” means *affirmative, conscious and voluntary agreement* to engage in sexual activity (according to the 2014 law). Meaning, when a person says “no” at any kind of sexual engagement, the other person must understand the “no” as such. Sexual contact with a person who has not given her/his consent constitutes a crime. The affirmative consent law “yes means yes,” includes three important elements: (1) the definition of consent as “*an affirmative consent standard in the determination of whether consent was given by both parties to sexual activity.* “*Affirmative consent*” *means affirmative, conscious, and voluntary agreement to engage in sexual activity*.” (2) The configuration of the sexual crime: it is understood that when a person says “no” at any kind of sexual engagement, the other person must understand the “no” and as such, the opposite reaction constitutes a crime. (3) It also marks the responsibility of the person who has to ensure consent: “*It is the responsibility of each person involved in the sexual activity to ensure that he or she has the affirmative consent of the other or others to engage in the sexual activity*.”

The change from “no means no” to the message of “get consent” caused several scholars analyze how young adults conceptualize consent (Beres, 2014). Worried for sexual violence prevention, education, and research on sexual consent, Beres studies the understanding of consent, from a perspective based on “communication about sex” and not only “ok sex.” This paradigmatic change makes the issue of consent greater than words.

Campaigns such as the one of PlannedParenthood^8^ defined consent as, such act either tacit or explicit, which involves the following criteria: (1) *Freely agreed*. Consent is a choice without pressure, manipulation or under the influence of drugs or alcohol. (2) *Reversible*. Anyone can change his or her mind about what they feel at any time; considering silence is not consent. (3) *Informed.* Anyone can only consent to something if he/she knows the full story of the facts and intentions. (4) *Enthusiast.* When it comes to sex, someone has to do what she or he wants to do, not the things that the other person might be expecting. (5) *Specific.* Saying yes to one thing does not mean a yes to other things or other people. While these definitions are essential when conssidering sexual consent understanding and training, the regulation of consent is indeed crucial.

As any other crime, sex crimes based on consent need to be considered, justified and properly formulated. But, why is consent relevant for sexual freedom? Historically, consent has been an important issue in social, economic and personal relationships. Consenting to a contract or a medical intervention is a legally recognized act based on the will of any human being. However, sexual consent would not always have the same relevance. According to Pérez Hernández (2016), a person could “formally” consent to having a sexual relationship or to sexual conduct (even saying “yes”) and not “really” want to participate in it, expressing their “decision” through words or silence.

Similarly, later movements show that “silence is not consent” (Spark movement, 2013^9^). Portugal passed its consent-based rape legislation^10^ following the “silence is not consent” principle. In these terms, silence is not be legally interpreted as consenting. Research also indicated some reasons that may affect someone’s own will (Mead, 1934; Walker, 1997), including coercion, given in fear, or for pleasinganother, among others. In our terms, consent means actively accepting to participate in any sexual activity. Sexual activity without consent is considered rape or sexual assault and is legislated as such in some countries. However, there is still an unsolved problem, in which this article will focus: Those situations in which, even with a “yes” the real message and the real will of the person is “no.” Thus, the challenge states “beyond words,” to interpret the attitude, the will and possible coactions, fears or other elements of the context that might influence someone at a psychological level.

### Previous Steps to Approach Consent Legally

Legislation offers legal certainty, which provides a solid foundation for the judge when making a decision. Indeed, one of the greatest impacts of legislating a reality states on achieving, through law, the legal certainty. At the point when a judge must make a decision, he or she needs to know, onboth, the facts which occurred andunder which legal category these facts have to be framed. The classification that a judge attributes to a fact (e.g., rape, sexual assault) is such according to its corresponding legal type. Thus, the better a social phenomenon is defined, the more concrete and more restricted it is, and the better interpreted it will be. This leaves less space for a judge’s own interpretation. Personal understanding of the facts can even be subjective, including separate opinions. This happened during the conviction of “La Manada” case, in which the aggressors were convicted for abuse and not for rape, based on opinion. This case constitutes an example of the need for a common legal framework. If different judges have different opinions, the lack of legal certainty may potentially lead to an ambiguous and conflicting decision, in which the collective subconscious uses to prevail, including those ideas taken for granted, as the one of “who keeps silent, grants consent” (Tomás, 2003).

The lack of consent constitutes a crime, and it is therefore aggression. Researchers have addressed the issue of informed consent, collective unconscious and tacit consent (Tomás, 2003). Here. the author reveals how the idea of consent has been harbored in our minds, from Roman law to Common law, creating what is considered a collective legal unconscious. For example, the phrase “who keeps silent, gives consent” was not included in Roman law but ended up being configured later in time until the present. In this way, Tomás argues that Civil law, unlike other systemic human rights, has led to configuring silence through principles created over time, but not through legal norms. Back in history, it was during the Canon law when silence was taken as affirmative acknowledgment, referring to fathers’ lack of verbal consent, so that their daughters could become nuns without their permission. The fathers’ absence of verbal consent led to consider silence as a legal act, with value given only to affirmative consent. However, in Roman law, individual silence was not considered consent anymore.

Facing the dilemma of the legal interpretation of silence, findings in psychology have already shown the existence of certain situations and mental decisions, conscious or unconscious, that can affect a person’s behavior pattern (Kandel, 2018). The psychological shock may occur due to fear, panic, anxiety or other situations of power that psychology has already defined as causing the inability to speak and immobility (e.g., turn cold, freeze) (Gidycz et al., 2008). Rape is one of those situations. However, following the mere legal thought, judges who are not aware of these psychological effects, tend not to seriously consider certain situations in which the victim is simply not able to speak. The link between psychology and the law becomes crucial at this point. The dilemma about the interpretation of consent used to emerge from situations in which it cannot occur or be requested, for instance, harassment and violence (often arisen under coercion, under the influence of alcohol or other substances or in sporadic relationships). These are certainly the kind of situations in which consent and its regulation are most necessary.

The contradictory outcome in a sentence, as happened in the case of “La Manada,” provide serious consequences, not only for the survivor’s victimization, but also for the emergence of “other Manada’s.” Even in many Spanish schools, boys under 16 years old have created “mini-Manadas” to attack their female classmates. Thus, the reality of gang rapes, many of them produced in hook-up situations (Puigvert et al., 2019), require the need to build a legal type, basing crimes on the lack of consent for any sexual act. This would contribute to social impact in the following terms: (1) by providing legal certainty; (2) reducing judges’ subjectivity when sentencing; (3) contributing to transforming the collective legal subconscious; (4) leading to increase the number of complaints for sexual harassment (which is positive in terms of victims’ coming forward); and this, (5) driving to reduce the emergence of “new Manadas.” Interpretations must be based on standard legislation.

### Current Legislation on Consent

The Istanbul Convention, article 36, states that parties shall take the necessary measures to ensure *the following intentional conducts are criminalized: engaging in non-consensual vaginal, anal or oral penetration of a sexual nature of the body of another person with any bodily part or object; engaging in other non-consensual acts of a sexual nature with a person; causing another person to engage in non-consensual acts of a sexual nature with a third person^11^*. In this sense, the Convention includes that “*Consent must be given voluntarily as the result of the person*’*s free will assessed in the context of the surrounding circumstances*”.

How should legislation approach consent? How have other legislations done so? Starting with considering the difficulty to legislate non-verbal interactions, there are some laws that have made little approximations. The “no means no” legislation (2004) pioneered. Years later, the positive consent emerged from below. Campus student movement claimed that saying “no” is not enough, since there are situations in which a person cannot say “no”; but even then, she or he might not be consenting. Thus, the need for affirmative consent raised and the struggle for a law claiming “anything less than yes is no”; also known as the “Yes means yes” law, passed in 2014 in the State of California. One year later, in 2015, the State of New York passed it consent law, as “Enough is enough,” stating: “*New York State has the most aggressive policy in the nation to fight against sexual assault on college campuses. By standing up and saying* “*Enough is Enough*,” *we made a clear and bold statement that sexual violence is a crime, and students can be assured they have a right to have it investigated and prosecuted as one*.”

Considering the United States legislation trajectory regarding consent, another key factor in this sense, occurred in 2013, when the Campus Sexual Violence Elimination Act (SaVE Act) was a bill whose components incorporated as an amendment to the Clery act. The Campus SaVE Act updated the Clery Act by expanding the scope of this law in terms of transparency, accountability and education. In other words, encouraging reporting, response and prevention education requirements on rape, acquaintance rape, domestic violence, dating violence, sexual assault and stalking.

Other approximations, in New Zealand and Canada, include for instance situations of intoxication, sleeping or death meaning that the person does not have the capacity to consent (Crimes Amendment Act, 2005^12^; House of Commons Bill C-49,1992^13^). In the European Union, currently only 9 of the 28 EU member states include in their jurisdictions rape as sex without consent, either tacit or explicit: Ireland, United Kingdom, Belgium, Cyprus, Germany, Iceland, Luxembourg, Sweden and Portugal. Some others, including Spain, embrace consent under the concept of sexual assault and only recognize it when physical violence or intimidation takes place.

The German Criminal Code, section 179, considers sexual offense as *situations in which the victim does not suspect an attack, is defenseless, or makes a refusal to consent to the sexual act known either verbally or through his or her behavior* (e.g., *by crying or stiffening*). This is a communicative act providing important information for other legislation to include consent. The Luxemburg Criminal Code, article 375 defines the lack of consent as a rape crime. It states: *Any act of sexual penetration, of whatever nature, by any means whatsoever, committed on a person who does not consent, including using violence or serious threats by ruse or artifice, or abusing a person incapable of giving consent or free to oppose resistance, constitutes rape and shall be punished by imprisonment of five to ten years*. In 2018, Iceland’s Parliament^14^ passed a landmark bill which makes *sexual relations with someone illegal, unless you have their explicit consent. Under the new law, consent must be clearly and voluntarily expressed*.

The Belgian Criminal Code (Act 375) defines rape as: *any act of sexual penetration committed on a person who does not consent. Consent is deemed to be absent when the act is imposed by means of violence, force or by a trick, or if the victim is suffering from a physical or mental disability*. The United Kingdom law considers informed consent as *freely, by both partners, enthusiastically, every time and for every sexual act*. *An intoxicated person is legally unable to consent to sex and having sex with a person who is very drunk is rape or sexual assault*. Swedish law includes the requirement of consent regardless of whether there has been violence or threats, or has violated the situation of vulnerability of the victim. It included the concept of “negligent rape” and “negligent sexual abuse.” In 2017 Ireland^15^ include in its Criminal Law Act, the sexual offense as *an act which if done without consent would constitute a sexual assault*; considering situations when a person lacks the capacity to consent to a sexual act if he or she is, by reason of a mental or intellectual disability or a mental illness incapable of consenting (specifying concrete situations). The Cyprus Criminal Code, Section 144 includes consent by stating: *Any person who has unlawful carnal knowledge of a female, without her consent, or with her consent, if the consent is obtained by force or fear of bodily harm, or, in the case of a married woman, by impersonating her husband, is guilty of the felony termed rape.*

The Spanish Criminal Code defines rape under the presumption of aggression. It defines sexual abuse, sexual harassment and sexual assault as follows: sexual abuse (article 181): *who without violence or intimidation and without consent, performs acts that attempt against the freedom or sexual indemnity of another person;* sexual harassment (article 184), *as the one requesting favors of a sexual nature, for himself or for a third party, within the scope of an employment, teaching or service provision, continued or habitual, and with such behavior will provoke the victim an objective and seriously intimidating situation, hostile or humiliating*; sexual assault (article 178) as *the one that attempts against the sexual freedom of another person, with violence or intimidation*. Article 179 specifies: *when the sexual assault consists of sexual intercourse by vaginal, anal or oral route, or introduction of objects by one of the first two routes, the person responsible will be punished as a criminal of rape*.

Gender and law are academic disciplines both linked to social concerns. People ask now for legislation on gender issues. While attempting the legal involvement seems something of extreme gravity that requires immediate solution, it is also true that laws make us free and prevent many undesired social behaviors. That is why when the law fails, it sends a warning message to society, to not be compliant or trustful. Legislation changes people’s morality, and we have changed laws accordingly. Human consciousness and laws have to go hand in hand. In ancient Rome, the to rape the daughter of a tax-paying citizen was an offense because it was his property; only the father could consent for the daughter, showing the crime was against the father, not against the woman, regardless of her consent (Ted Talk, Joyce Short^16^).

In German law, acts such as crying and screaming are included, and some legislative developments do include non-verbal language in an attempt to legislate consent. However, the psychological perspective is crucial, as it is necessary for the other person to interpret the message as such. The case that Joyce Short explains demonstrates the interactive power, since the boy is the connoisseur of the information and therefore the one who has to ensure the free and equal dialogic interaction. The same video shows how, similar realities differ in different states of the United States, according to their rape or consent legislation. That is why it is necessary to go beyond current legislations.

In short, taking into consideration all the previous arguments and definitions on consent for any sexual relationship, it should be affirmative, agreed, free, informed, without coercion, based on the lack of interactive power and institutional power, extended from the beginning until the end of each sexual engagement, based on the non-verbal communicative acts. In this line, crimes on rape, sexual aggression and abuse should be treated based on consent or the lack of it.

## Consent From Speech Acts to Communicative Acts

How can we be certain when someone consents or not? Language is one of the channels. In a normal way of communication, people “tell” their will. Verbal language is key, but so is how it is understood and how it is applied. In this space, the role of psychology intervenes, the willingness to interpret, understand what the other person wants to tell and pretends to communicate, even without saying a word. Thus, the complexity of defining consent is not so much its definition, but its applicability (Katz et al., 2019). While there is a quiet widespread agreement on the way consent is defined, the debate is driven to know exactly what consent does involve and the context surrounding it (Muehlenhard et al., 2016). States of unconsciousness, alcohol, and drugs make a person unable to provide consent. In addition, fear, intimidation, power relationships, evaluations from professors to students, letters of recommendation, are example situations that restrict the “no” of the victim and even cancel it. Thus, positive consent arises, which also can be nullified at some point.

By accepting a “no” is to understand that the other person does not belong to us, even if even if he/she had consented at the beginning with a yes; even consenting without wanting to be there. The psychological debate used to be focused on the victim, in showing resistance, running, being afraid or calling out. On the other hand, the social debate must focus the aggressor, it must be clear that consent is a requirement for any kind of intimate act. In this vein, other existing research helps us to advance in the knowledge and the application of consent. Thus, the communicative acts and the researches on this topic, so far are key to be applied to the study and achievement of sexual consent.

In his theory on “Speech Acts,” Austin (1955) discussed *How to do things with words*, giving examples of how words create realities. For instance, by saying yes, a marriage is created. Austin also realized that language depends on some conditions. Illocutionary and perlocutionary speech acts focus on the intentionality of the speaker. John Searle, constituted his linguistic phenomenology in this vein. For Searle (1969), intentionality in context is key for any speech act. Thus, the “construction of social reality” for Searle (1995) is based on intentionality too, which is not only for the individual but also collective society. Its speakers share this collective intentionality. For example, a five-Euro bill is a five-Euro bill because people have agreed so.

Along these lines, Habermas (1987) published his “Theory of communicative action,” in which he used the concept of communicative action. Searle and Soler (2004) talk about “dialogic communicative acts,” in which, both the context and the consequences of our communicative action are important in the development of the action, due to its influence in the construction of a wide range of social phenomena (Searle and Soler, 2004). Soler Gallart (2017) introduced and analyzed the relevance of non-verbal communication (gestures, easy expressions, tone of voice, etc.). That is, not only do “we do things with words” but also with non-verbal symbols, which can communicate by themselves, or be accompanied by words. In this way, body language also “says” a lot and so, communication is not just about talking, but inquiring about the context in which communicative acts take place.

From the social psychology perspective, Mead (1934) developed the symbolic interactionism proposing the external world as the place where the subjective world is constructed. The external and the internal worlds are both interacting and shared by people in the social world. Meaning is creating through language, in this process of intersubjectivity. Thus, the Habermas approach of communicative action focuses on social interaction for people understanding, beyond language communication. The act of speech is based on interactions. Among the different types of actions defined by Habermas, communicative action arises as an ideal type because language functions as a means to achieve an essential understanding to reach consensus and to take action among people.

## Materials and Methods

This article is based on a theoretical reflection and the transferability criteria, framed in line with communicative acts and their link to gender-based violence. The authors have been developing their research on this for years. This review presents evidence that have been found under the framework of communicative action and the prevention of GBV in light of consent and how legislation on consent can be enabled. We put all this knowledge at the service of two aspects: those situations in which consent may happen and conversely those situations in which consent does not exist. New steps focus on contributing to create a legislative framework able to build future legislation.

We also analyze, first, the definition and theoretical advances made so far on the issue of consent., and then, different existing legislations on consent in both the United States and Europe to present here. Beyond these studies, the article involves an analysis of cases recently occurred and published in different media: (1) the case of La Manada^17^; (2) the gang rape case of Manresa (Barcelona)^18^; (3) a case raised by Joyce Short during her Ted Talk^19^ *;* and (4) a case presented in the section of the “New York Times Opinion”^20^. Research in social psychology provides the necessary framework for analyzing communicative acts based on symbolic interaction, that is, on Mead’s theory (1934) describing the link between self and society, which leads to a constant dialogue between the person and her or his self, as responsible for the self-consciousness.

Assuming that none of the girls whose cases are analyzed here consented to what occurred, this article studies which situation, intervention, legislation and/or measure would have improved the consequences of their situations. Based on the criteria of transferability, among actions and interactions, we will focus on those cases which are transferable to other contexts. In this way, several verbal and non-verbal communicative acts that lead someone to raise awareness of others’ response regarding consent or the lack of it, will be enumerated. Here the will of the other person to interpret the communicative act and their psychological disposition to the facts are raised (Mead, 1934).

Therefore, one of our goals is collecting these transformative communicative acts (eye looking, criteria to act, ways of responding to third parties’ coercion, etc.) and explain them with examples of cases on the Internet and the media in Spain and in the United States. These actions may not only transform someone’s present, but they could also change their future by aiding the reconstruction of people’s autobiographical memories of their worst life episodes in healthy directions (Racionero-Plaza et al., 2018).

This kind of methodology, analyzing data and cases by considering people’s voices (Puigvert et al., 2017) have already being used by several research projects and published in respected journals showing social impact and transformation (Gómez González, 2019). The whole idea of the need for training in any specific area, such as the need for bystander training, has also led us to appreciate the importance and the requirement to educate into consent, to raise awareness about it, all in all to contribute to the overcoming of gender-based violence. Data of this research will be analyzed twofold, on one hand based on the *Social Impact Open Repository* (SIOR^21^) and on the other, based on the European Commission study *Monitoring the Impact of EU Framework Programmes^22^*.

SIOR was created as one of the outcomes of the IMPACT-EV framework project of research, constituting a tool that enables researchers to share the social impact of their own research projects with other researchers as well as with stakeholders (Flecha et al., 2015). SIOR established five criteria for evaluating political and social impact: (1) connection to United Nations Sustainable Development Goals, EU2020 targets or other similar official social targets; (2) percentage of improvement achieved in relation to the starting situation; (3) replicability of the impact: the actions based on the project findings have been successfully implemented in more than one context; (4) publication by/in scientific journals (with a recognized impact) or by governmental or non-governmental official bodies; (5) sustainability: the impact achieved by the action based on project findings has showed to be sustainable throughout time.

Drawn on the societal impact, the report on monitoring the impact of the European Framework Program for research and innovation, elaborated by experts, established a set of indicators, divided into short-term, medium-term and long-term indicators, following four key impact factors: (1) addressing global challenges; (2) achieving Research and Innovation mission; (3) engaging EU citizens; (4) supporting policy-making. In this line, the following set of indicators for the societal and policy key impact pathways includes considering: the difference between outputs and results; the estimated cost necessary for their collection; the knowledge and transference concepts to determine social impacts; the level of reporting burden for beneficiaries; and the impact timeframes.

## New Approach to Consent

Current advances in the study of communicative action point out to the issue of linguistics, involved in symbolic interaction and the creation of the worlds around us, which can be strongly connected through analyzing the predominance of dialogic relationships regarding power interactions, based on the following four points: (1) institutional power; (2) interactive power; (3) consequences and intentions; (4) regulation vs. prohibition.

### Institutional Power

Institutional power refers to that which usually exists within institutions influencing their organigram and hierarchy. In the context of universities, it may be embodied by professors who have, at least, symbolic power over students, in some vulnerable situations such as, grades, the ability to decide on their academic future, recommendation letters. In this case, consent could not be asked nor given, since institutional power might limit or prevent the student’s freedom to reject or to say “no” to his or her professor. To name another situation, institutional power may exist in a company context. Companies also have power structures that characterize the way they function. There are high, low and medium managerial positions. To the extent that some managerial positions rule over others, it attributes more power to highest charges. In this way, for example, if a boss asks a secretary to have a beer after work, the freedom of that person in a lower position can be diminished by the power of the other person. If harassment occurs while having that beer, consent cannot be given nor requested, since the difficulties for ensuring that it is actually voluntary and free.

### Interactive Power

Interactive power refers to that power provided by the interactions established among people. For instance, one classmate could threaten a girl with sextortion (Patchin and Hinduja, 2018) if she does not say “yes” to having sexual relationships with him. Another example: five boys with a girl in a small doorway, besides the normal interaction being spoiled, there is an additional kind of power established by the interaction itself. In relation to consent, the group of five men or just one must know that, in that interaction, they have more power, because of the context. Considering the desire to have a sexual relationship with the girl, they have to be very sure about getting consent; as otherwise, if the girl would complain at some point, society will stand on her side. That is, the most vulnerable person in the relationship would get the social support. Under this scenario, interactive power is determined by context, which provides more power to one person over another. For instance, if two friends decide to have dinner in the house of one of them, the host has more power that the guest, just because of the context. If a blurry line surrounds consent, position has to accompany consent, because of its weaker position.

### Consequences/Intentions

Weber (1930:1905) defined the ethics of responsibility referring to the consequences and not the intention of any action committed. In this case, following the ethics of consequences involves considering whether the consequence of the action conducted between two or more people has been the desired one, or the contrary of what happened. Although the intention might have been a good one, a male boss inviting a woman candidate to go to a pub, during the selection of unemployed people for a job, the consequence of that fact could be that she feels pressed to say “yes.” In this sense, “good intentions” do not justify “bad consequences” or the outcome desired by all the people involved. Regarding consent, ensuring the best-intended outcome continuously is the duty of all people involved into action. Providing another example, let us imagine someone convicted for aggression, declaring something that, “I didn’t want to harm her” at the beginning of the dispute. In that case, the fact to be judged is the consequence of the matter, not the initial intention, but the final consequence. In this scenario, consent needs to be assured until the end, in other words, the consequence is crucial to determine if consent occurred or not.

### Regulation vs. Prohibition

This model involves both situations, the regulation and the prohibition of any potential sexual-affective relationship under a context based on institutional power, as for instance, the academia. It does not necessarily mean that relationships between professors and students are not allowed, or between five boys and a girl should be considered a crime; as a power relationship, even when conducted between adults, they are under the power system. To find solutions to this dilemma, some of the highest ranking universities have decided to prohibit and condemn all and any kind of sexual relationships between a professor and his/her students. Other high-ranking universities have taken the option to allow those potential relationships while they are freely consented, and both members inform the university. However, if the most vulnerable later complaints, the university will take his/her side. This scenario contributes to the field of consent by considering situations in which, even current legislation allows consent in a sexual relationship (because of age), there are situations under which that relationship may not be free. However, in order for it to be so and for no one to be harmed, situations where interactive power and institutional power have to be regulated; also in order to allow, and not necessarily prohibit, a relationship between a student and a professor or between an employee and a boss; while also believing the most vulnerable person.

## Analysis

The results of this study are configured based on four cases already mentioned above: (1) the so-called gang rape, La Manada; (2) the case of Manresa; (3) the rape described in the Joyce Short’s Ted Talk; and (4) the situation explained in the opinion section of The New York Times, in which consent seems to be given but the lack of both sides sharing the same information, convicted him for rape^23^. They all are different stories with common elements. The public information released about these cases included episodes of young women who did not give their verbal consent, as well as cases of girls whose lack of negation may have been understood as consent, but they did not want to participate on any of the acts occurred. Above all, these examples highlight the need to address consent also from a non-verbal perspective, beyond words, the essential for training on non-verbal communication, as well as to raise awareness on others’ influence on decisions., Mead described this through his theory on social interaction creating ways of being, thinking and acting. University needs to consider prevention, not just punishing the issue of consent and its consequences, but being aware that this problem constitutes a public health issue.

### The Case of La Manada

For the last 2 years, the Spanish people have watched the most controversial trial for a rape case. Five men started talking to a girl, who was sitting alone on a city bench. Subsequently, they put her in a doorway, and all began to rape her in different ways, one after another. In the end, they stole her cell phone and left her there alone. This happened in July 2016 during the celebration of the Pamplona’s regional party, San Fermín. In April 2018, the five aggressors were sentenced for sexual abuse and not for rape. The issue was consent: the 18-year-old victim did not say “no.” Rape is not configured based on consent, under the Spanish Penal Code, and one of the judges had a particular vote. That same day thousands of people were demonstrating in the streets in favor of the survivor. Social pressure was necessary, both to demonstrate a step forward as a society, supporting survivors and a way of pressing for the change of current legislations. We all took her side, we believed her, and mostly citizens defended her right to not show negation; we understood her fear to be killed in case she dared to say no. In June 2019, the Supreme Court sentenced La Manada for rape, and raised the punishment to 15 years in jail. This ruling established doctrine on intimidation, and considered this enough reason to break victim’s will. Nevertheless, the criminal reform is still incomplete, and concepts such as harassment, aggression and violence need to be adjusted, to provide legal certainty to the legislator and prevent particular opinions.

### The Manresa Case

At the beginning of July 2019, the trial against six men began. Five men directly raped a young woman aged 14 years old in 2016, including intercourse and sexual acts while she was unconscious. A sixth man already knew the victim, according to the public prosecutor report. He was completely aware of her age, and saw that she was barely conscious, after drinking alcohol and smoking marijuana. He took her to a nearby location and allegedly raped her. After that, he encouraged his friends to do the same while the girl was unconscious. They have been accused of raping the minor in an old factory of Manresa, in Barcelona. The victim, who is now 17, reported that she was raped in a building that night. The men were arrested and convicted by the public prosecutor with the lowest charges for sexual abuse, which could be raised to sexual aggression. Again, the issue for the judicial debate is consent. The victim testified her fear while observing a gun during the aggression. Her horror to be killed, justifies her lack of negation. This case should be link to a similar crime in Spain. We all remembered Nagore, a 20-year-old woman who was killed during the Pamplona festival in July 2008. She had accepted –coerced by her friends– to go to a boy’s apartment, but she said no to sex and was killed.

### Joyce Short’s Ted Talk

Universities are also spaces where sexual harassment occurs. After much research and much progress in relation to harassment based on power relationships, it turns out that harassment also occurs among peers, and that is where consent covers a crucial relevance. However, some examples demonstrate how the consent line is blurry and how getting consent needs to take into account contextual situations and minor nuances. In this sense, Joyce explains in this Ted Talk the case of a young man who entered, late at night, into a young woman’s room in their university dormitory. Though asleep, she felt someone get into her bed. Thinking itwas her boyfriend, engaged in sex with him. The act could have beeen consensual at some point, but it was not here because she did not have the full information. Thus, the police analyzed the case and arrested him for rape. The issue of informed consent is raised here, as only he had all the information. For these additional contextual reasons, it is necessary to delve into this problem in order to expand its circumstances, as well as to clearly delimit those situations under which consent cannot be agreed or requested.

### The New York Times Opinion Case

Hanna Stotland argues that simply expelling college students accused of sexual assault is a misguided response to what is a public health problem. In her video, Stotland describes very different cases surrounding consent and the difficulties to get it right, with different degrees of what is considered sexual assault while she is asking to name each action by a different term. During the video she explains a case of a man and woman who both agreed to have a sexual relationship. After a while she filed a complaint against him based on the lack of consent. She recognized that she said yes at the beginning, but later, said she did not mean it at that time, but just agreed to have sex in order to leave the room more gracefully. The man was accused for rape and was suspended from the university for 2.5 years. According to Stotland, this example make us aware that there are confusing moments when yes might actually mean no, so consent is a murky process and universities should look for justice while training students to navigate this gray zone for prevention of raped, and not being accused of rape.

## Results: Transformative Communicative Acts

There is a broad agreement on the definition of consent, which has to be affirmative, voluntary, enthusiastic, conscious, and repeated. The issue is of how to get it in the right way, and how to punish someone when he or she did not get consent. Communicative acts and dialogic interaction are a contribution to this regard. Lidia Puigvert (El Diario Feminista, 2019) already describes some prerequisites for a consenting relationship. For example, the turn from relationships based on speech acts to the ones of communicative acts, which include all types of communication not just the verbal. It means to base the relationship on dialogic interactions and not on power interactions, some of which may come through power manifested by institutions, for instance a boss over his female worker. However, considering that power interactions may exist in the absence of institutional power, interactive power should be considered. For example, five boys with one girl in a doorway.

Referring to the cases of La Manada or the Manresa gang rape, it is proven that there was a sexual act in which women recognized being coerced. When a group of men is alone with a woman, they should know that she might not feel free. So, they only can try to have any kind of sexual contact if they are sure that it is a totally free relationship; knowing that they are taking the risk if they are wrong. There is much concern about educating in consent. Consent education involves creating collective awareness, both about the severity of violence and the importance of society taking position against this problem. The consequences of GBV can become unbearable psychologically. This human grief needs to be addressed from a scientific perspective, even when research is still wondering how, each new step leads to a new reality, still unsolved. GBV is an emerging and urgent issue for scientists who, from psychology, seek ways to impact it.

Drawn from our research, based on SIOR and Monitoring impact criteria, the following set of actions point out as being transformative in order to add to sexual consented relationships, while contributing to the social impact of physiological research. The concept of consent needs to include:

(1) **Ethics of responsibility.** Accounting for power interactions in an unequal social structure. Limitation of the idea of consensus proposed by Habermas based only on validity claims and orientation toward understanding.
(2) **Non-verbal body language.** This is crucial, as it does makes little sense to to ask at every moment “do you want to keep doing this?”
(3) **Provide conditions free of coercion.** Conditions that enable consent means ensuring spaces and interactions in which consent is freely given, clear, continuous, specific and unambiguous. Situations of duress, power relationships, unconsciousness, fear and threat, cannot ensure consent.
(4) **Solidarity with survivors.** In any situation when someone fills a complaint for sexual harassment, everybody’s duty is to believe survivors and be in solidarity with them. In the same way, this action involves empower and protect active bystanders.
(5) **Consent training.** The need for asking for and getting consent should be trained, speaking about its challenges but also its benefits.
(6) **Communicative acts, beyond words**, need to be considered for ensuring consent for any sexual activity. Nobody should ever judge a victim for the way she or he reacted once sexually assaulted.
(7) **Common sense.** Some legislations are based on tradition, jurisprudence and common sense. In a moment when legislations on consent are being built, situations in which the meaning of consent is not clear (verbally and non-verbally), common sense may be used.
(8) **Overcome barriers and resistances.** While achievement of consent for any free sexual relationship is not easy, local and structural barriers should be considered and overcome.

## Discussion

### Evidences of Social Impact of Psychology

In May 2019, at the Oñati International Institute for the Sociology of Law, took place the Workshop^24^ on GBV including a roundtable discussion on the issue of consent, of which the authors have taken part discussing with members of the police, the issue of gender violence, its link to consent, and the need to add this approach to their cases. Additionally, we shared this contribution with lawyers, scholars, representatives of women lawyers’ associations, gender experts, policy makers and social workers as well as with survivors and educators. They all could appreciate the social impact of this research to the reality they are facing each day.

Based on current approaches to consent, there are two clear scenarios, so far. The “no means no” and the “yes means yes,” following the principle “anything less than yes is no.” However, there is a third situation, to which this article aims to contribute, considering occasions when “yes,” a potential “yes” or even a silence, actually means “no,” referring to those situations in which a specific context pushes the person to have no choice but to say yes (or to agree). Following this line, to approach specific contexts, we build knowledge along analytical elements in two veins: (1) the communicative acts and the will for understanding them from a psychological perspective; (2) the interactive power and the institutional power, which frame specific contexts. Consequently, new realities create on us, as researchers, the duty to provide scientific elements both for the analysis of cases as well as for legislating them. Some of these realities are described in section 6, such as the case of “La Manada,” The “Manresa case, The Joyce Short’s Ted Talk, and The New York Times” Opinion Case. These cases show the need to consider consent in a conscious manner, from the begging of the engagement until the end, and informing at every moment the partner about the intentions. In the same line, the way consent has been taken into account from a legislative perspective; it shows the importance of analyzing these realities as a pressing moment for the creation of new legislations in relation to consent. This section presents below, three cases that influenced legislation in their own countries of origin. Usually, legislation need reality first in order to be created and changed.

### Three Cases That Impacted Legislation

Previous studies have already shown the importance of the gang rape analysis and it trials in terms of what is considered social opportunity, that social moment necessary to raise awareness and contribute to make possible translating a social claim into a law with the aim of legislating on affirmative “yes” (Vidu and Tomás Martínez, 2019). As for instance, without the struggle for women’s rights, we would not have legislation about it. Laws shape our morality; we need new laws on sexual assault to change the way people think and act regarding to them.

(1) As history show us, specific cases have promoted legislations on sexual harassment**. *Clery Act*** or the *Jeanne Clery Disclosure of Campus Security Policy and Campus Crime Statistics Act* is a federal law passed in 1990 as a consequence of the Jeanne Clery rape and murder in 1986 by another student, at her campus university residence hall. At that moment, it was discovered that 37 other cases involving violent crimes occurred at that university during the last 3 years. This is how the ***Clery Act law*** requires institutions to disclose, publish and distribute their data and statistics on violent episodes.
(2) The Portuguese legislation on consent was passed in January 2019, once the Third Criminal Section of Lisbon has confirmed a 6 years and 6 months prison sentence for aggravated rape to a man aged 35 who in September 2016 took a girl aged 14, without her consent and raped her without her resisting. According to the judicial sentence, the judge considers that the absence of physical resistance of the victim cannot be considered a form of consent, but a tool to survive the attack.
(3) The Chanel Miller’s rape case (known as Emily Doe during the legal process of the complaint) also changed the California law. She was raped while unconscious at her campus in 2015. One year later, Chanel was reading the victim-impact statement in a courtroom in California. The statement was later published on BuzzFeed^25^ and had more than 18 million views. The Chanel case triggered a change in the California law, which at that moment did not consider her case as rape. Currently, the definition of rape into law includes any kind of penetration and there is a mandatory 3-year minimum prison sentence for penetrating an unconscious or an intoxicated person^26^.

The three cases presented above are clear examples of the issues of responsibility, body-language interpretation, providing situations free of coercion, solidarity with survivors, common sense and training on consent. Linking these points with the four cases presented in section 6, highlighting the impact of psychology in any and each of them. For instance, in “La Manada” case, once the men knew she is helpless, they are five, she is alone and feared, they even did not ask her about consent as they do not want to know her will. The psychological point is skipped from acting in accordance with the will of others, in line with what Mead says. In the case of Manresa, the men were aware that she is unconscious, they really want to rape her, and they do not want her consent. Psychologically, the men are aware of what they do. In the case of the Ted Talk, at the time he knew that she is not his boyfriend, he knew that he is cheating on her. In the same line, for the New York Times’ Opinion case, she said yes at the begging but actually meaning “no,” so interpretation beyond words is needed, and communicative acts arises in order to better understand her willing.

### Increasing Movement of Supporting Survivors

All these cases share the need and the difficulty to define and legislate sexual consent. The cases of La Manada and the Manresa case were also in the media and were deeply rejected by the feminist movement after the provisional request of the Prosecutor’s Office which accused them of sexual abuse instead of sexual assault awaiting the victim’s testimony. Under the slogan *We do believe you*, massive support for both victims and rejection of harassers was publicly shown. In this sense, La Manada as a case study for this article, shaped the proper legal opportunity to build new legislation on the issue of consent, including context of the action and its features. This is a historical moment for lawmaking on sexual harassment and consent. Through legislating different contexts and situations which may occur, it will be possible to better prevent GBV and harassment. The social impact serves to raise a debate in the social and legal field, feasible to overcome victimization and contribute to the effective use and achievement of sexual consent. It is necessary to advance, beyond words, into the interpretation of silence, considering the interactive power as well as the institutional power.

Along the same lines, there are already programs that have led people to act differently. The *It*’*s on* United States campaign^27^, says: *non-consensual sex is sexual assault*. Here it becomes necessary to stablish situations which make consent ineffective, as power, force, duress or deception. However, we still need to establish how actually consent is implemented. Some campaigns define it as: *freely given, knowledgeable and informed agreement*. In her web page^28^ on consent awareness, Joyce Short makes a difference between *assent* and *consent.* Permission is a form of assent, but consent has a different meaning according to the law. This legal distinction makes some sexual conduct, even those containing assent but not consent, to be criminal. According to the Anti-Violence project^29^, the “no means no” messages of the 1990s have been replaced with “yes means yes” and “consent is sexy” messages particularly for use in poster campaigns and slogans used in “slut walks” as examples. There is also increased focus on consent in a range of anti-sexual violence education programming. “Consent it’s as simple as tea”^30^ is a campaign consisting on describing what consent is for all ages; being specially based on the idea that consent can be given and ungiven during the same sexual conduct. In her Ted Talk video^31^, Amy Adele Hasinoff, talks about what “sexting” teaches us about consent. While digital communication has positive effects, affirmative consent needs to be simple. She discusses mutual communication and the simple and clear response when someone asks for consent from the other person, really gets it. Most importantly, she agrees that speaking about consent for decades now, makes people more aware on the fact of asking and getting consent, but specially, on the consequences of not getting consent.

Considering the findings of psychology, legislating consent will provide juridical information for legal certainty, contributing to right interpretation of unconsented sexual encounters. Severe legal basis, including psychological reactions disabling consent, will increase victim protection and complaints, while contributing to reduce the emergence of gang rape cases.

## Conclusion

While rape is not always a problem of miscommunication, and consent is still complicated to be defined under the law spectrum, the contribution of communicative acts and dialogic interactions is unprecedented in the research in psychology and its impact for society. Psychology has already contributed to impact this issue from its previous research, highlighting Mead’s symbolic interactionism and the communication among people based on consensual dialogue.

In the same line, considering communicative actions and egalitarian dialogue for consenting sexual affective engagement is certainly apioneer important contribution. Indeed, interactive power beyond structural power opens a new channel to understand situations for which the current definitions of consent have shown not to be broad enough to respond to current realities. The dialogic sessions we have had with relevant lawyers, police officers, gender specialists, educators, social workers and victims have outlined the clear social impact of this line of research.

Day by day society is more demanding and needs more answers to current problems. It is time to eradicate GBV. In our duty to provide scientific knowledge to this claim and achieve the goal of contributing to preventing aggressive sexual contacts from early ages, we suggest people’s relationships might be based on communicative acts and consent established as a space in which dialogic interactions can be freely asked and given.

## Author Contributions

All authors equally contributed to conceived the presented idea and discuss it with members of others fields of study, gender specialists, policemen responsible of gender violence cases, and policy-makers implementing prevention and response mechanisms and directly working with survivors and have deeply debated on the psychological perspective of consent, on categorizing its legal frameworks and analyzing concrete contextual situations. RF contributed to the conceptualization of the reality of sexual consent and to gestate the interactive and institutional power in analyzing the context. GT contributed to develop thoughts on the legal analysis and the social and legal advancement of the consent notion. AV contributed to the formal analysis and discussion and the writing of that part of the manuscript.

## Conflict of Interest

The authors declare that the research was conducted in the absence of any commercial or financial relationships that could be construed as a potential conflict of interest.

## Footnotes

1 For more information, see: http://www.catalannews.com/society-science/item/three-men-arrested-for-gang-rape-in-seaside-town

2 For more information, see: https://www.theguardian.com/world/2019/aug/08/five-french-men-arrested-on-suspicion-of-gang-rape-in-costa-blanca

3 For more information, see: https://www.bbc.com/news/world-europe-49248954

4 For more information, see: www.geoviolenciasexual.com

5 For more information, see: https://www.adultdevelopmentstudy.org/

6 For more information, see: https://ec.europa.eu/sustainable-development/goal5_en

7 For more information, see: NSVRC. 2015. National Sexual Violence Resource Center. Available at: https://www.nsvrc.org/sites/default/files/publications_nsvrc_factsheet_media-packet_campus-sexual-assault.pdf

8 For more information, see: https://www.plannedparenthood.org/planned-parenthood-st-louis-region-southwest-missouri/blog/blog-post-about-consent

9 For more information, see: http://www.sparkmovement.org/2013/03/15/silence-is-not-consent-silence-is-the-problem/

10 For more information, see: https://www.reuters.com/article/us-portugal-rights-rape/portugal-approves-law-widening-definition-of-rape-idUSKCN1P51MQ

11 For more information, see: https://rm.coe.int/168008482e

12 For more information, see: http://www.legislation.govt.nz/act/public/2005/0041/latest/DLM346175.html

13 For more information, see: http://publications.gc.ca/site/eng/115148/publication.html

14 For more information, see: https://www.thejournal.ie/iceland-consent-3943673-Apr2018/

15 For more information, see: http://www.irishstatutebook.ie/eli/2017/act/2/enacted/en/print

16 For more information, see: https://www.youtube.com/watch?v=imr5ZiAY_ao&t=636s

17 For more information, see: https://www.bbc.com/news/world-europe-44569918

18 For more information, see: https://elpais.com/elpais/2019/07/08/inenglish/1562585604_503153.html

19 For more information, see: https://www.youtube.com/watch?v=imr5ZiAY_ao&t=636s

20 For more information, see: NYT opinion section: https://www.nytimes.com/2019/07/09/opinion/sexual-assault-students-campus.html?action=click&module=Opinion&pgtype=Homepage

21 For more information, see: https://crea.ub.edu/fecyt_sior/acerca-de-fecyt-sior/

22 For more information, see: https://publications.europa.eu/en/publication-detail/-/publication/cbb7ce39-d66d-11e8-9424-01aa75ed71a1

23 For more information, see: NYT opinion section: https://www.nytimes.com/2019/07/09/opinion/sexual-assault-students-campus.html?action=click&module=Opinion&pgtype=Homepage

24 For more information, see: http://www.iisj.net/es/workshops/la-superaci%C3%B3n-de-la-doble-pobreza-de-las-mujeres-v%C3%ADctimas-de-violencia-de-g%C3%A9nero

25 For more information, see: https://www.buzzfeednews.com/article/katiejmbaker/heres-the-powerful-letter-the-stanford-victim-read-to-her-ra

26 For more information, see: https://www.latimes.com/politics/la-pol-sac-stanford-rape-prison-sentences-20160806-snap-story.html

27 For more information, see: https://www.itsonus.org/

28 For more information, see: https://consentawareness.net/2016/01/31/assent-vs-consent-theyre-not-one-and-the-same/

29 For more information, see: https://avp.org/

30 For more information, see: https://www.youtube.com/watch?v=oQbei5JGiT8

31 For more information, see: https://www.youtube.com/watch?v=LdDRv2f2dFc
